# A Comprehensive Review of Deep Learning Applications with Multi-Omics Data in Cancer Research

**DOI:** 10.3390/genes16060648

**Published:** 2025-05-28

**Authors:** Flavio Sartori, Francesco Codicè, Isabella Caranzano, Cesare Rollo, Giovanni Birolo, Piero Fariselli, Corrado Pancotti

**Affiliations:** Computational Biomedicine Unit, Department of Medical Sciences, University of Torino, Via Santena 19, 10126 Torino, Italy; flavio.sartori@unito.it (F.S.); francesco.codice@unito.it (F.C.); isabella.caranzano@unito.it (I.C.); cesare.rollo@unito.it (C.R.); piero.fariselli@unito.it (P.F.); corrado.pancotti@unito.it (C.P.)

**Keywords:** deep learning, cancer genomics, multi-omics, drug response, survival analysis

## Abstract

The integration of deep learning (DL) with multi-omics data has significantly advanced our understanding of biological systems, particularly in cancer research. DL enables the analysis of high-dimensional datasets and the discovery of novel disease mechanisms and biomarkers, contributing to improved patient treatment and management. This review provides a detailed overview of recent developments in deep learning models applied to genomics data, with a focus on cancer type classification, driver gene identification, survival analysis, and drug response prediction. We introduce the foundational concepts of machine and deep learning and explain the characteristics of multi-omics data, addressing a broad and interdisciplinary audience. Methods published since 2020 are systematically reviewed, including their model architectures, datasets, and key innovations.

## 1. Introduction

The genomics field has experienced transformative advancements with the integration of machine learning (ML) techniques, which have allowed researchers to interpret vast and complex genomic datasets, uncovering previously unknown patterns and insights. By efficiently processing large-scale data, ML algorithms have proven their ability to identify genomic biomarkers, significantly enhancing our understanding of disease mechanisms and allowing for new possibilities in personalized medicine and treatment strategies [1,2].

The rapid progress in high-throughput sequencing technologies has generated an extraordinary volume of genomic data, facilitating the development of ML methodologies across various biological domains [3]. In cancer genomics, ML applications have achieved substantial success across multiple levels. For example, ML has been fundamental in identifying mutational signatures [4,5,6,7,8]—distinct mutation patterns derived from whole-genome (WGS) and whole-exome sequencing (WES) data that are linked to specific etiologies [9,10]. Routine analysis of mutational signatures now enables patient stratification and supports research in highly personalized therapy settings. By identifying characteristics such as homologous recombination (HR) or mismatch repair (MMR) deficiency, clinicians can implement more targeted, individualized treatment approaches [11,12].

Furthermore, Deep Learning (DL), a subset of ML that utilizes neural network architectures, has demonstrated remarkable versatility and precision in analyzing heterogeneous biological data across multiple omics layers. Techniques such as convolutional neural networks (CNNs) [13], graph neural networks (GNNs) [14], recurrent neural networks (RNNs) [15], and Transformer-based networks [16] have revolutionized multi-omics data integration and interpretation. These computational approaches have been successfully applied to numerous cancer research challenges, including classification of differentially expressed genes [17,18], patient stratification based on molecular profiles [19,20,21], survival analysis prediction [22,23,24,25], somatic mutation identification from genomic data [26,27], and identification of epigenetic signatures associated with cancer progression [28,29].

Neural networks are becoming increasingly popular and valuable in the analysis of histopathological images [30,31,32]. Recent studies have demonstrated significant advancements in extracting meaningful morphological patterns from cancer tissues, which can also be linked to molecular features such as tumor mutational burden (TMB), RNA expression, and deficiencies in MMR and HR related genes, with promising perspectives [33,34,35]. This progress holds great potential for clinical practice, as histopathological imaging analysis offers a cost-effective alternative to WGS and WES techniques.

While single-omics (e.g., genomics) exploitation has demonstrated its ability to classify tumor types, identify new biomarkers, and build accurate disease phenotypes, the integration of multi-omics data has gained significant importance to uncovering insights and relationships that cannot be highlighted when each omic layer is considered alone. This comprehensive view has the potential to strengthen the foundation for precision medicine, allowing for a thorough analysis of patient disease and facilitating treatments tailored to individual molecular profiles [36,37,38].

Beyond oncology, ML-driven multi-omics have shown important advancements in a broad range of biological applications. In cardiovascular and neurodegenerative diseases [39,40,41,42], for example, ML methods have been applied to integrate genetic data with epigenetic, transcriptomic, and even environmental information to uncover risk factors and implement novel therapeutic targets. As research continues, machine learning’s ability to process and interpret multidimensional data is likely to drive breakthroughs in diagnostics and treatment, potentially changing how multiple complex diseases are managed.

**Figure 1:**
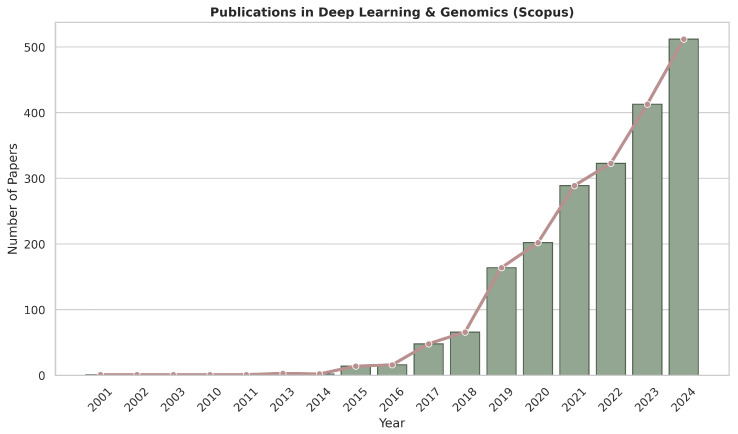
Trend of publications in deep learning and genomics.

**Figure 2:**
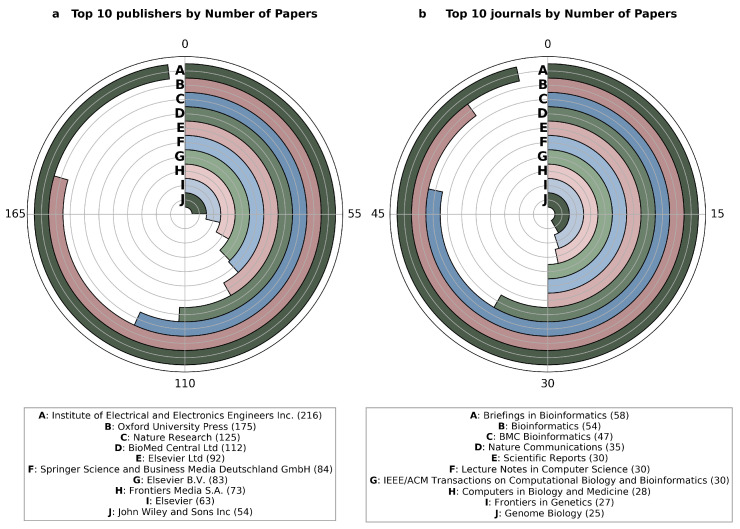
Comparison of the distribution of deep learning publications among publishers (**a**) and the source journals (**b**).

In recent years, the number of publications on deep learning in the field of genomics has steadily increased, as illustrated in Figure 1 and Figure 2. Given the availability of comprehensive reviews already present in the literature—and to avoid substantial overlap—this work will focus specifically on deep learning methodologies applied to cancer genomics from 2020 onward.

This review aims to provide the reader with a comprehensive overview of the fundamental principles and best practices in machine learning and deep learning, along with an introduction to various multi-omics data types.

We then explore key applications of integrating multi-omics data with deep learning, with a focus on four main topics:

- Cancer type, subtype, and unknown primary origin classification;
- Driver gene prediction and discovery;
- Survival analysis;
- Drug response prediction.

We aimed to adopt an objective criterion for selecting the papers. Specifically, as mentioned above, we focused on deep learning papers published from 2020 onward. Given the wide range of available works and the significant overlap between models, we initially selected papers based on the number of citations. To ensure we did not overlook more recent publications, we also considered those present in high-impact-factor journals.

While this review does not offer a direct comparison of methods, it presents a systematic overview of current methodologies, emphasizing the techniques employed, the types of data utilized in their implementations, their strengths, and possible limitations.

## 2. Basic Concepts and Principles in Machine Learning and Deep Learning

### 2.1. Types of Learning

Machine learning can be categorized into supervised, unsupervised, self-supervised, and reinforcement learning. Each of these approaches has different characteristics and applications.

- **Supervised Learning**: supervised models are trained on labeled data, which means that each data point is associated with a known output. The model learns how to map the inputs to their corresponding outputs, enabling it to generalize the acquired knowledge to new data. A biological example of supervised learning is predicting whether a DNA sequence with a particular mutational pattern is associated with a specific disease. The model is trained on sequences labeled as disease-associated or not, and it will learn the characteristics that differentiate the two groups.
- **Unsupervised Learning**: This deals with unlabeled data, so the main tasks are related to uncovering hidden structures within the data. The algorithm, through the maximization of similarity measures, identifies intrinsic patterns, without any supervision. In genomics, unsupervised learning can be applied to tasks such as clustering tumor samples based on their gene expression profiles, thus allowing for the identification of molecular subtypes. Unsupervised methods can group genes with similar expression patterns under different conditions, which may reveal insights into underlying biological processes or disease mechanisms.
- **Self-Supervised Learning**: This is a form of learning where the model generates its own labels from the data, rather than relying on provided labels. It often involves predicting parts of the input from other parts of the same input. This type of learning is useful when there is a large amounts of unlabeled data available. In biology, a self-supervised learning approach could involve predicting missing parts of a genomic sequence based on the context, helping the model learn useful representations of the data without requiring explicit annotations.
- **Reinforcement Learning**: This focuses on training agents to make a sequence of decisions by interacting with an environment. The model learns through trial and error, receiving rewards or penalties based on the actions taken. In a biological context, reinforcement learning can be applied to protein folding. A reinforcement learning model could explore different configurations of a protein sequence, receiving rewards when it folds into a more stable or functional conformation. This process mirrors how biological systems optimize complex tasks through iterations, such as how cells might improve the efficiency of molecular interactions or enzyme catalysis over time based on feedback from the system’s state.

**Figure 3:**
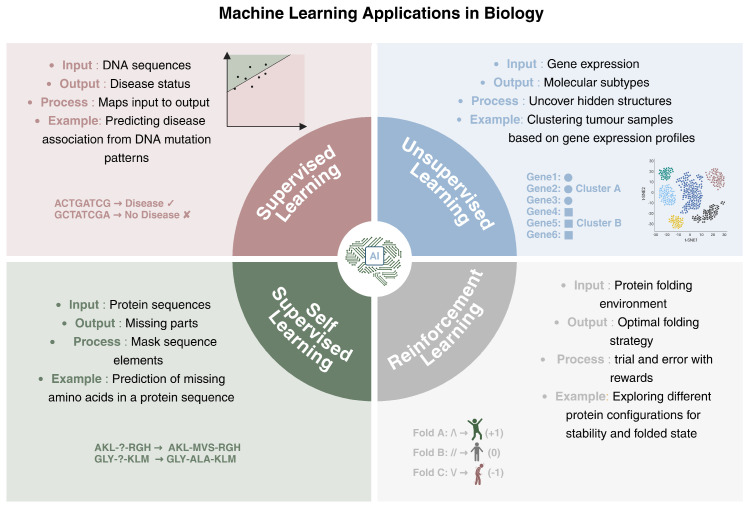
**Different types of learning**. Some basic examples of machine learning applications in biology.

Figure 3 provides a visual summary of different types of learning with applications in biology.

### 2.2. Best Practices

When developing a machine learning solution, it is important to follow some specific principles to ensure the algorithm’s robustness and to guarantee its ability to generalize to new, unseen data. These practices are fundamental to achieving the best possible model designed for solving a specific task.

- **Preprocessing**: This is a crucial step that ensures data quality and consistency before model training. It typically involves data cleaning—handling missing values through imputation or filtering—and features preprocessing techniques such as normalization, standardization, and encoding categorical variables. Dimensionality reduction methods like PCA [43], t-SNE [44], and autoencoders [45] are often applied to mitigate the curse of dimensionality and to enhance model performance. In the context of genomic data, common practices include initial quality control—such as assessing sample integrity, read quality, and detection rates—to remove low-quality samples or features. This is followed by correcting for batch effects [46,47,48], filtering low-expressed genes in RNA-seq data, and normalizing expression levels. In DNA methylation analysis, data are often preprocessed through background correction, normalization, and filtering of low-quality probes [49,50]. For multi-omics integration, preprocessing involves aligning heterogeneous data types—such as transcriptomics, epigenomics, and proteomics—through techniques like feature harmonization and scaling, ensuring that combined signals reflect meaningful biological relationships while reducing any source of noise or bias.
- **Metrics**: based on the preliminary preprocessing analysis, the nature of the problem, and the dataset, choosing the most suitable metric significantly changes the reliability and effectiveness of the implemented machine learning method. For classification tasks, common metrics include area under the curve (AUC), precision–recall area under the curve (PRAUC), sensitivity, specificity, F1-score, and Matthews correlation coefficient (MCC). To properly measure performance, it is usually good practice to provide a complete overview of the metrics, as some of them can be better suited (e.g., AUC, accuracy) to unbalanced datasets. For a complete overview and definitions of classification metrics for both binary and multi-class classification scenarios we redirect the reader to [51,52].
  For regression tasks, common evaluation metrics include Mean Squared Error (MSE), Mean Absolute Error (MAE), Pearson correlation (*r*), and the coefficient of determination ($R^{2}$). These metrics assess the accuracy and variance explained by the model. Please refer to [53] for mathematical formulations of the common regression metrics.
  For survival analysis, key evaluation metrics include the Concordance Index (C-index), which quantifies the agreement between predicted and actual survival outcomes; the time-dependent Area Under the Curve (AUC), which assesses model discrimination at specific time points; and the Integrated Brier Score (IBS), which evaluates overall prediction accuracy across the entire follow-up period. A complete review of survival metrics can be found in [54]
- **Train–Test Split and Cross-Validation**: a common practice is to split the available dataset into training and test sets (e.g., 80/20). The training set will be used to effectively train the model and to optimize it, while the test set will serve to evaluate the model’s ability to accomplish the given task on points never seen during the training time. In particular, the training set is further divided using the k-fold cross-validation strategy. This technique estimates the model’s generalization error by dividing the training set into *K* subsets. K−1 folds are used for training, while the remaining fold is used to validate the prediction by calculating a specific evaluation metric. This process is repeated *K* times, ensuring that each fold is used as a validation set once. The average validation error across all iterations provides a robust estimate of model performance. Cross-validation is commonly employed for hyperparameter tuning, but in the absence of an external blind test set, it can also serve as a surrogate for model evaluation, providing an estimate of generalization performance.
  However, depending on the nature of the problem and the specific task, it might be beneficial to use stratified K-fold cross-validation instead of a standard K-fold approach. This is particularly useful when dealing with imbalanced datasets, where certain classes are underrepresented. In a traditional K-fold split, the distribution of classes in each fold may not be representative of the overall dataset, potentially leading to biased training and evaluation. Stratified K-fold addresses this issue by preserving the class distribution within each fold, ensuring that each subset maintains a proportionally similar representation of the different categories present in the dataset. This is crucial in classification tasks, as it prevents the model from overfitting to the majority class while underperforming on minority classes.
- **Hyperparameter Tuning**: each ML algorithm (e.g., random forest, support vector machines) has specific hyperparameters (e.g., number of trees, penalty) that should be optimized to obtain the best-performing model. During the cross-validation, a range of hyperparameter values is tested to determine the configuration that optimizes the user’s predefined metrics. This ensures that the selected model is the optimal one for the given task based on the desired metrics.
- **Testing and Generalization**: once the optimal model is identified, its final evaluation is performed on the test set, which was not used during training. This step measures the model’s ability to generalize to new data, ensuring it can obtain accurate predictions in real-world data.
- **Unbiasness**: bias and fairness remain critical challenges in machine learning [55], often arising from different sources such as imbalanced datasets, measurement inconsistencies and annotation errors. In the field of biology, for example, limited genetic diversity in reference databases or the under-representation of rare diseases can lead to biased models that fail to provide accurate predictions for minority groups. Fairness-aware algorithms, such as adversarial debiasing [56], tackle these issues by training models to minimize reliance on sensitive attributes, such as demographic factors, while maintaining high prediction accuracy. Additionally, cross-population validation studies enable models to be evaluated on diverse and independent population groups, ensuring that predictions generalize effectively. By combining these approaches with techniques like oversampling rare cases [57] and reweighting underrepresented data, it becomes possible to achieve robust and reliable performance in biological applications.
- **Explainability**: understanding the challenges of explainability in machine learning is essential, particularly in the context of biology and clinical applications. One significant challenge is the difficulty of explaining how models capture biological mechanisms and how predictions can be interpreted, especially for large datasets. Additionally, the transparency of highly accurate methods, such as deep learning, is often limited, while interpretable models like decision trees may lack the performance needed for complex tasks [58]. These limitations come from the way our brains work, making it hard for us to fully understand how models make decisions or what factors influence them [59]. Despite these challenges, significant advances have been made in developing tools like SHAP [60] and LIME [61]. These model-agnostic tools improve transparency by providing approximations of how models make decisions. They work by explaining the model’s behavior and identifying the factors that influence its predictions. Through techniques like visualizations and feature importance scores, these tools help users to understand which inputs are most impactful, making it easier to trust and understand the model’s decisions and ensuring safety in critical applications. In tasks involving image data—such as histopathology or medical imaging—techniques as saliency maps [62], Grad-CAM [63], and attention weight visualization have become valuable. These methods highlight the regions in an image that most contribute to the model decision, offering intuitive insights into model behavior. Through such visualizations and feature importance scores, these tools help users understand which inputs are most impactful.

**Figure 4:**
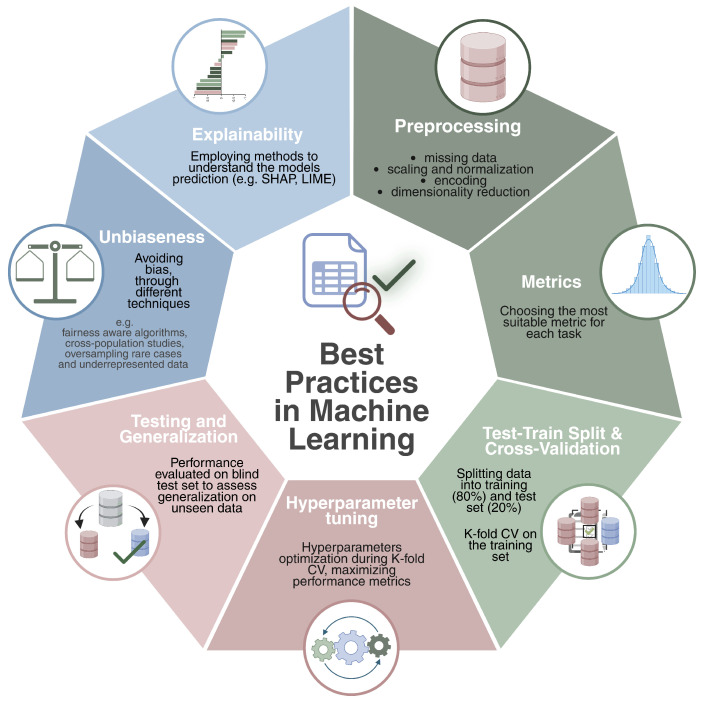
**Best practices in machine learning**. The diagram illustrates the essential components of a robust machine learning pipeline, including preprocessing, appropriate metric selection, train-test splitting with cross-validation, hyperparameter tuning, testing and generalization, ensuring unbiasedness, and model explainability. Each component contributes to building reliable, fair, and interpretable ML models.

The points discussed above are not meant to be fully comprehensive, but they aim to highlight the importance of following well-established practices when developing a machine learning tool. In Figure 4, we summarize all of the presented concepts.

### 2.3. Deep Learning

**Figure 5:**
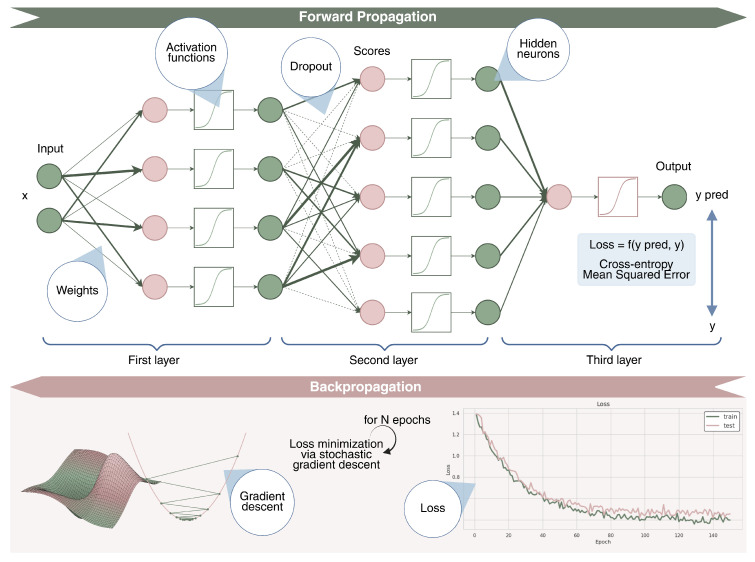
**Deep learning model architecture and training workflow**. Overview of the key components and workflow of deep learning models, highlighting their layered structure, training process via forward and backpropagation, use of loss functions to quantify prediction errors, and optimization using Stochastic Gradient Descent (SGD).

Deep learning is a branch of machine learning that leverages neural networks to learn from data and make predictions. Neural networks consist of layers of nodes, or neurons, which process information in a hierarchical manner. The input data are passed through consecutive layers, where each neuron performs a weighted sum of its inputs, followed by an activation function. This process is called forward propagation. The network’s goal is to map the input to the correct output, such as a class label (e.g., tumor types) or a regression value (e.g., I50 value). During training, the network adjusts its weights to minimize a loss function, which measures the difference between the predicted and actual outputs. For classification tasks, common loss functions include cross-entropy loss, while for regression, mean squared error and its variants are often used. The process of adjusting weights is done through the backpropagation [64], where the gradients of the loss function are computed and used to update the weights using an optimization algorithm like Stochastic Gradient Descent (SGD) [65] and its variants [66,67]. This process repeats over multiple iterations (epochs), allowing the network to improve its accuracy. To prevent overfitting (i.e., memorization of the training data without proper learning), regularization techniques like dropout [68] or L2 regularization can be used, helping the model generalize better to new, unseen data. In Figure 5, we provide a visual understanding of the presented concepts.

In biology and bioinformatics, deep learning has become a powerful tool across a variety of domains due to its capacity to model complex, non-linear relationships in high-dimensional data. Different neural network architectures are applied depending on the data type and biological question. For instance, GNNs and GCNs are well-suited for modeling molecular interaction networks, such as gene–gene or protein–protein interactions, by capturing topological and relational structures. CNNs are widely used in histopathological image analysis, enabling automated detection of cancer subtypes, tissue classification, and cellular feature extraction from whole-slide images. For genomic sequences, Transformer-based and attention mechanisms are becoming increasingly popular due to their ability to model long-range dependencies in DNA, RNA, or protein sequences, offering both high performance and interpretability.

## 3. Introduction to Multi-Omics

Cancer is a highly heterogeneous disease, marked by complex genetic, epigenetic, transcriptomic, proteomic, and metabolic alterations that are responsible for tumor initiation and progression. In recent years, research has increasingly focused on the integration of these biological layers to gain a more comprehensive understanding of tumor biology. Within this framework, different omics technologies are fundamental in cancer research, each offering distinct and complementary insights into the molecular architecture of the disease.

- **Genomics** focuses on the comprehensive analysis of the genetic landscape of cancer cells, with the goal of identifying mutations, structural variations, and alterations that drive tumor initiation and progression. Technologies such as Whole Genome Sequencing (WGS) and Whole Exome Sequencing (WES) are fundamental for profiling both coding and non-coding regions of the genome. These approaches enable the detection of somatic alterations including Single and Multiple Nucleotide Variants (SNVs, MNVs), insertions and deletions (indels), Copy Number Variations (CNVs), and more complex structural events. Additionally, genomic data are used to assess Microsatellite Instability (MSI), which reflects defects in the DNA mismatch repair system, and to study others genomic properties that can help to better profile cancer.
- **Epigenomics** investigates chemical modifications of DNA and histones that regulate gene expression without altering the underlying DNA sequence. Epigenetic changes, such as DNA methylation, histone modifications, and chromatin remodeling, are responsible of controlling gene accessibility and transcriptional activity. These modifications affect gene silencing, activation, and cellular differentiation, and they are often dysregulated in cancer. Aberrant epigenetic patterns can lead to the inactivation of tumor suppressor genes or the activation of oncogenes, contributing to cancer initiation and progression. Key epigenomic techniques include DNA methylation analysis (e.g., MeDIP-seq [69] and Bisulfite sequencing [70]), histone modification profiling (e.g., ChIP-seq [71]), and chromatin accessibility assays (e.g., ATAC-seq [72]), all of which help to understand how chromatin structure and gene expression are altered in cancer.
- **Transcriptomics** provides insight into gene expression patterns by analyzing RNA molecules, offering a snapshot of how genetic information is transcribed and regulated. Key methods in transcriptomics include RNA Sequencing (RNA-seq [73]) for global gene expression profiling, Long Non-Coding RNA (lncRNA-seq [74]) for the study of non-coding RNAs involved in gene regulation, MicroRNA (miRNA-seq [75]) to identify small RNAs regulating mRNA stability and translation, and single-sell RNA-seq (scRNA-seq [76]) for profiling gene expression. Additionally, spatial transcriptomics allows for the mapping of RNA expression within tissue architecture, providing insights into the tumor microenvironment.
- **Proteomics** is the comprehensive study of the full set of proteins produced in a cell, tissue, or organism, focusing on their expression levels, structural modifications, and interactions. It provides valuable insight into cancer research by providing information on how protein dynamics change during cancer development and progression. One of the key technologies in proteomics is mass spectrometry, including methods like tandem mass spectrometry (MS/MS [77]) and liquid chromatography–mass spectrometry (LC-MS [78]), which enable precise identification and quantification of proteins in samples. Proteomics also allows for the mapping of protein–protein interaction networks, which are essential for understanding the molecular pathways involved in cancer. A critical aspect of this field is the investigation of post-translational modifications such as phosphorylation, ubiquitination, and acetylation, which influence protein function and are often dysregulated in cancer.
- **Metabolomics** studies the metabolic alterations in cancer cells, analyzing small molecules involved in cellular metabolism. Techniques such as Nuclear Magnetic Resonance (NMR [79]) spectroscopy and LC-MS are used for the profiling and quantification of intracellular and extracellular metabolites, providing insights into tumor metabolism, progression, and drug response.
- **Lipidomics** explores the lipid composition of cancer cells, examining the role of lipids in cancer metabolism and progression. By characterizing lipid profiles through techniques like LC-MS technology, Gas Chromatography–Mass Spectrometry (GC-MS) [80], and shotgun lipidomics [81], it is possible to uncover the significance of lipid metabolism in tumor biology.
- **Microbiomics** examines the role of the microbiome in cancer development and therapy response, highlighting the importance of microbial communities in the tumor microenvironment. 16S rRNA sequencing and metagenomics (shotgun sequencing) are used to profile the microbiota associated with tumors, providing insights into how the microbiome may influence cancer progression and therapeutic outcomes.
- **Interactomics** investigates molecular interactions, such as protein–DNA, protein–RNA, and protein–protein interactions, to map the regulatory networks driving cancer. Techniques like ChIP-seq [82] and CUT&RUN [83] are used to analyze protein–DNA interactions, while CLIP-seq [84] and RIP-seq [85] explore protein–RNA interactions. Protein–protein interactions, mapped through methods like Yeast Two-Hybrid (Y2H) [86] reveal the complex networks of molecular interactions driving cancer biology.
- **Imaging omics** merges medical imaging and molecular data, extracting quantitative features from imaging modalities (e.g., H&E, CT, MRI, PET) and integrating them with molecular data for a more comprehensive understanding of tumor behavior. The use of AI to analyze digital pathology slides is at the forefront of this field, enabling more accurate cancer diagnosis and prognosis.

The integration of these omics technologies, combined with machine learning approaches, is revolutionizing cancer research. By creating a more comprehensive understanding of tumor biology, these multi-omics approaches hold promise for more precise diagnostics, prognostic biomarkers, and personalized therapeutic strategies for cancer patients.

**Figure 6:**
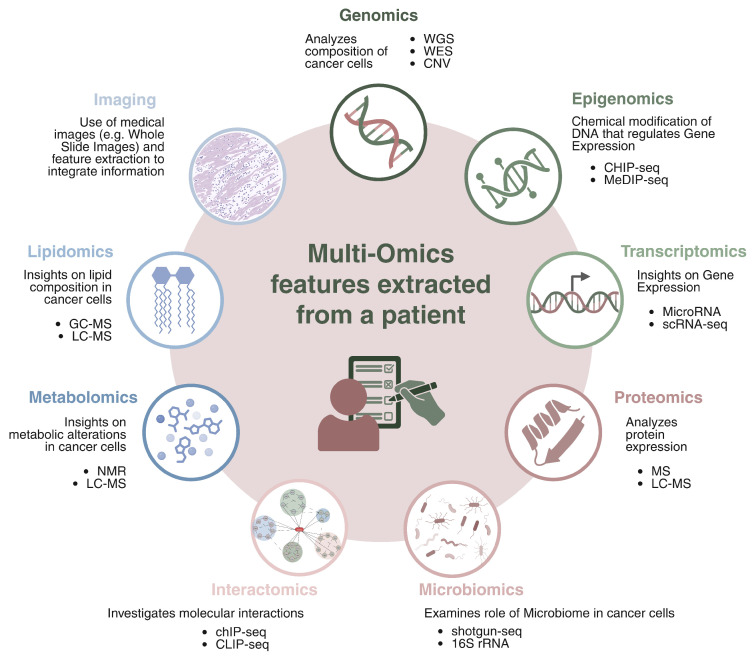
**Overview of multi-omics features extracted from a patient**. The diagram presents various omics layers used to characterize cancer, including genomics, epigenomics, transcriptomics, proteomics, metabolomics, lipidomics, microbiomics, interactomics, and imaging. Each omics type provides unique biological insights through different technologies and contributes to a comprehensive understanding of disease mechanisms.

Figure 6 provides a visual summary of the omics information that can be extracted from a patient.

## 4. Deep Learning Applications in Cancer Omics

### 4.1. Classification of Tumor Types, Subtypes, and Unknown Primary Origin

Molecular subtyping of tumors is fundamental in precision oncology, as it enables the classification of cancers based on molecular characteristics rather than only on histopathology. This stratification facilitates more accurate diagnosis and improve prognostic assessment by identifying patterns associated with disease etiology and clinical outcomes. However, the complexity and high dimensionality of molecular data present significant challenges. In this context, deep learning has emerged as a powerful tool, modeling non-linear relationships within large-scale datasets, offering the potential to enhance the identification of tumor types, subtypes, and even infer the origin of cancers of unknown primary origin (CUP). CUP accounts for approximately 3–5% of all cancer diagnoses and is associated with poor outcomes due to the lack of site-specific treatment strategies. Accurate molecular classification in such cases could significantly improve patient management by suggesting likely tissue origins and enabling customized therapies.

Among the deep learning based tools, **DeepType** [87] was developed to address limitations in existing tumor subtyping methods [88,89] and to uncover biologically meaningful subtypes with high-dimensional genomics data. In particular, supervised learning approaches focused on separating predefined classes, often ignoring genes that could reveal novel subtypes. On the other hand, unsupervised clustering methods risk identifying clusters based on features alone, not incorporating prior biological knowledge, limiting their relevance to cancer biology. Hence, the key innovation of DeepType is the integration of both supervised and unsupervised learning in its framework. The model uses gene expression profiles from approximately 20,000 genes and implements a feed-forward neural network trained with cross-entropy alongside a K-means loss function. The K-means loss function is used to direct influence the latent space, such that the compressed representation of cancer samples can have better separation in distinct groups.

Validation experiments were conducted on breast cancer data from the METABRIC [90] cohort and bladder cancer data from the TCGA [91] cohort. In the BRCA cohorts, for instance, the method selected 218 genes and grouped the data into 11 distinct clusters, including 10 tumor-specific clusters and 1 cluster for normal tissue samples. Using t-SNE visualization, these clusters were shown to be well-separated, with strong alignment to the established PAM50 [92] subtypes. In addition, the survival analysis highlighted the clinical importance of these subtypes, showing distinct prognostic outcomes with strong statistical confidence. Each subtype had unique transcriptional profiles tied to specific gene co-expression modules, including luminal A, luminal B, and *HER2*+/basal subtypes. This molecular diversity went beyond the PAM50 classification, providing more detailed insights into breast cancer taxonomy.

Yang et al. introduced **Subtype-GAN** [93], a multi-omics-based generative adversarial network designed as a multi-input, multi-output framework for tumor subtyping. Subtype-GAN was trained and validated on data from ten tumor types (BRCA, BLCA, KIRC, GBM, LUAD, PAAD, SKCM, STAD, UCEC, and UVM) obtained from the TCGA database. The architecture of Subtype-GAN features three key components: an encoder module consisting of a series of independent layers for processing each omics data type separately (copy number, DNA methylation, miRNA, mRNA) and a shared layer that represents the common latent representation of tumor samples; a decoder module that reconstructs the original omics data from the latent space through the independent layers; and a discriminator module ensuring that the latent space follows a prior Gaussian distribution by “penalizing” samples that are far from it. Additionally, the framework incorporates a Gaussian Mixture Model (GMM) module to determine the optimal number of molecular subtypes from the learned latent space. The authors validate Subtype-GAN through a proof-of-concept analysis of BRCA multi-omics data from 1031 samples. Specifically, they identified five clusters corresponding to well-established breast cancer subtypes (e.g., basal-like, luminal-A). In addition, they observed significant differences in the survival curves across the five clusters (*p*-value = 5 × $10^{-3}$). Among the clusters, basal-like ones exhibited the longest average survival time, followed by luminal-A and luminal-B-like clusters. In contrast, normal-like and *HER2*-enriched clusters were associated with poorer prognoses, aligning with previous knowledge about breast cancer subtypes [94]. Subtype-GAN also identified five key biomarkers (*ERBB4, FOXA1, SLC26A9, GJB3, TFF3*) associated with known cancer pathways, further confirming the reliability of the results.

**DICLR** [95] was developed by Cai et al. It consists of an advanced variational autoencoder framework enhanced with a contrastive loss function [96] and self-supervised clustering. The main model innovation is the ability, reached through a dedicated network module, to disentangle noise from meaningful biological signals in multi-omics data and to extract consistent representations, which are critical for accurate cancer subtype prediction. DICLR is built upon the reliable assumption that each omics dataset comprises a latent consistency variable, which is relevant for clustering, and noisy variables that are biologically irrelevant. The model was trained and evaluated across ten cancer types from the TCGA. In particular, for each cancer, two evaluation criteria were used to assess the reliability of the results. (1) A log-rank test was used to calculate the P-value of survival analysis curves to test if significant differences existed between the identified subtypes, and (2) a Chi-Square test and the Kruskal–Wallis test were used to check the enrichment of clinical variables (e.g., age at diagnosis, gender, metastasis) in the predicted subtypes. The results showed that DILCR identified subtypes with significant differences in survival patterns and strong enrichment of clinical features, supporting the validity of its classifications. To further validate the biological relevance of the model, DICLR subtypes identified in BRCA and KIRC datasets were analyzed in more detail. For BRCA, the identified subtypes were compared with established molecular characteristics and PAM50 RNAseq classifications. Notably, two subtypes identified by DICLR showed strong alignment with the Basal-like group, which is characterized by negative expression of ER, *HER2*, and PR. Another DICLR subtype appeared to be heterogeneous, containing samples from multiple known subtypes. Meanwhile, the two remaining subtypes overlapped with both Luminal-A and Luminal-B, likely reflecting their biological similarity.

DICLR was also evaluated on the KIRC dataset, reavaling four distinct subtypes with significant differences in gene expression profiles. Differentially expressed genes such as *EGFR, ESRRG, ALDOB*, and *BIRC5* were identified, which are supported by previous cancer studies [97,98].

Recently, Sanjaya et al. developed **MuAt** [99], an attention-based deep neural network used to predict tumor types from cancer PCAWG [100] and TCGA [91] whole-genome and whole-exome sequencing data from 24 different tumor types, achieving prediction accuracy of 89% for whole genomes and 64% for whole exomes and top-5 accuracies of 97% and 90%, respectively. MuAt leverages DNNs’ ability to learn representations in a supervised setting with the attention mechanism, allowing the model to focus on relevant mutation sequencing data elements, improving performance and explainability. Specifically, MuAt integrates single-nucleotide and multi-nucleotide substitutions (SNVs/MNVs), short insertions and deletions (indels), structural variant (SV) breakpoints, and combinations of these genetic alterations. By learning multimodal data embeddings, it represents mutation type and genomic position at a per-mutation level, allowing the model to learns a fine-grained somatic mutation representation. In fact, MuAt demonstrates the ability to distinguish tumor subtypes that were not explicitly provided as input. These include tumors influenced by somatic and germline mutations, such as prostate cancers with somatic *SPOP* mutations and pancreatic endocrine tumors with germline *MUTYH* mutations. It also identifies hypermutable subtypes, like microsatellite-unstable cancers and polymerase proofreading-deficient tumors. MuAt utilizes its attention mechanism to derive factors that correlate strongly with established mutational signatures, aligning these factors with tumor-specific characteristics. These attention-derived factors link patterns of single-base substitutions (SBS), doublet-base substitutions (DBS), and indels (ID) to well-known mutational processes. For instance, the model identifies factors corresponding to UV-induced damage, reflected in SBS7 and ID3 in skin melanomas, and to tobacco smoke exposure, associated with SBS4 and DBS2. Similarly, factors connected to homologous recombination deficiency correlate with SBS3 and related indel patterns. This approach allows MuAt to represent mutational processes in a way that complements traditional signature analyses, providing a biologically informed understanding of the genomic landscape of tumor types. To validate the results, the model was also applied to whole-genome sequences from the ICGC cohort that were not part of the PCAWG, and to whole-genome cancer sequences from Genomics England (GEL) [101]. This dataset includes thousands of cancer genomes across 23 tumor types. For benchmarking, they selected the seven tumor types that matched those in PCAWG. Finally, MuAt was validated on an independent cohort of colorectal cancer whole genomes that were also not used during training. In all of these datasets, MuAt demonstrated state-of-the-art performance, confirming the generalizability of the method across diverse cohorts. This highlights its robustness as a framework, further enhanced by the attention mechanism, which enables the identification of mutations specifically associated with particular tumor types and subtypes.

As previously noted, accurate tumor-type identification is fundamental for clinical decision-making in cancer, as it guides therapy selection and clinical trial eligibility. Despite the proven capability of the current genomic-based classifiers, they often rely on whole-genome or whole-exome sequencing (WGS/WES), RNA-seq, and other type of sequencing depending on the type of inputs, which are costly, lack scalability, and are unavailable in routine clinical settings.

To address these limitations, the **Genome-Derived-Diagnosis Ensemble (GDD-ENS)** [102] was developed by Darmofal et al. using data from MSK-IMPACT [103], a targeted cancer gene sequencing panel profiling over 500 genes. MSK-IMPACT is widely accessible and has already been used to sequence over 75,000 patients, making it a practical basis for a large-scale genomic classifier. GDD-ENS improves upon earlier models by incorporating deep learning and expanding the number of tumor types from 22 to 38, covering 97% of solid tumors in the cohort.

The model is an ensemble of neural networks trained on around 40,000 tumor samples, with features derived from mutations, copy-number changes, structural variants, mutational signatures, and tumor mutation burden. GDD-ENS achieves an average accuracy of 78.8% and macro-precision of 64.2%, improving to 87.0% and 75.8% when including the second-highest prediction, and 90.2% and 78.1% with the third-highest. The main innovations of the method lie, first, in the use of an ensemble of ten neural networks, each independently optimized on different validation folds. This design allows the method to be particularly flexible with a better calibration and capable of adapting to out-of-distribution data. Secondly, despite relying on a limited number of panel-based mutations, the method achieves accuracy comparable to approaches based on whole-genome or whole-exome sequencing (WGS/WES). This represents a valuable solution for clinical management.

Validation experiments have demonstrated that GDD-ENS generalizes well to other targeted sequencing panels because most of the genes covered by MSK-IMPACT overlap with those in other large cancer panels. This makes it easy to integrate GDD-ENS into existing clinical workflows, even in settings where other assays are used. Finally, the GDD-ENS model also incorporates features for detecting rare tumor types and cancer of unknown primary origin (CUP), thus expanding its utility in challenging diagnostic scenarios.

Vibert et al. [104] developed the **TransCUPtomics** classifier, a machine learning tool based on RNA-sequencing data and a variational autoencoder (VAE) to predict the tissue of origin (TOO). The method was trained on 20,918 samples covering 94 diagnostic categories, including 39 cancer and 55 normal tissue types. The variational autoencoder encoded the high-dimensional transcriptomic data into a 100-dimensional latent space, which were consequently fed into a random forest classifier. The final model was validated on retrospective and prospective cohorts of CUP patients, achieving a 96% accuracy on reference data. The predicted TOO was identified in 79% of CUP cases, enabling tailored therapies that led to notable clinical responses and improved patient management. UMAP visualization of the latent space showed clear clustering of tumor and normal tissue types, with CUP samples aligning to specific diagnostic groups. For CUP predictions, high-confidence diagnoses required consistent results across RF and KNN models, yielding actionable insights in 67% of cases. A minority of samples (21%) remained unclassified, often due to unique transcriptomic profiles not represented in the training dataset. The tool supports CUP diagnosis and therapy optimization, offering a significant step forward in improving clinical outcomes.

**DeepTumour** [105] is a deep learning model developed by the PCAWG consortium to predict the tissue of origin of tumors using only somatic passenger mutations derived from whole-genome sequencing (WGS) data. A peculiarity of the model is that it was trained only on passenger mutations excluding driver ones, which were found to offer no additional benefits to the prediction accuracy. The model was trained on WGS-derived features from 2606 tumor samples from ICGC and TCGA cohorts spanning 24 cancer types and validated using external datasets of primary and metastatic tumors collected in [106] and [9], respectively. The architecture consists of a standard feed-forward neural network that incorporates a wide range of genomic features, including SNVs, indels, CNVs, and structural variant breakpoints, as well as sample-level information such as purity, ploidy, and the distribution of mutational event types across chromosomes.

The PCAWG model demonstrated robust performance across both internal and external validation cohorts, achieving an accuracy of 91% on held-out tumor samples and 88% and 83%, respectively, on independent primary and metastatic samples of external cohorts, establishing it as an innovative method for identifying cancers of unknown primary origin. Interestingly, the authors also provide a user-friendly web interface https://deeptumour.oicr.on.ca/submit (temporarily offline as of 27 May 2025) that allows users to upload a VCF file and receive automated tissue-of-origin predictions.

To address the diagnostic complexity of CUP, the **TOAD** [32] model was developed for predicting both the site of tumor origin and metastatic status from haematoxylin and eosin (H&E)-stained histology slides. The deep learning-based model was developed utilizing a huge dataset of 32,537 whole-slide images collected from public repositories and the Brigham and Women’s Hospital, including 18 common primary cancer types. TOAD utilizes a weakly supervised multitask learning framework, incorporating attention-based multiple instance learning [107] to identify diagnostically relevant regions within each slide. TOAD achieved, on the held-out test set of tumors with known primary origins, a top-1 accuracy of 83% and a top-3 accuracy of 96%, whereas on the external test set, it achieved top-1 and top-3 accuracies of 80% and 93%, respectively.

The model peculiarity is that it uses a weakly supervised multiple instance learning framework where each whole-slide image (WSI) is treated as a bag of patch-level features extracted by a pretrained ResNet50. These features are aggregated using a multitask attention-based pooling mechanism, followed by fully connected layers and a late-stage fusion with patient metadata (e.g., sex, age) for the final multitask classification of tumor origin and metastatic status.

Another important feature of the model is its interpretability. Attention heatmaps are generated during inference, and they visually indicate the tissue regions that influence the model’s predictions. In a curated cohort of CUP cases, TOAD predictions matched the differential diagnosis assigned by clinicians in 61% of cases using the top prediction and in 825 when considering the top three predictions. In summary, TOAD presents a scalable and accessible diagnostic tool, particularly valuable in settings with limited access to molecular testing.

A summary of the methods for tumor classification can be found in Table 1.

### 4.2. Driver Gene Prediction

Cancer driver genes are critical in tumor development, as their mutation or aberrant expression drives cancer cell growth [109,110]. Identifying these genes is essential for understanding cancer pathogenesis, patient prognosis [111,112], and developing targeted therapies [113,114]. In recent years, many computational methods have been developed to identify driver genes, each based on different assumptions. Frequency-based methods [115,116,117] assume that driver genes tend to have more mutations, so they focus on genes that are more frequently mutated. On the other hand, network-based methods [118,119] see cancer as the result of changes in several genes that interact with each other and affect biological pathways. Each approach has its own drawbacks. Frequency-based methods often miss genes that are rarely mutated but still important. Network-based methods depend heavily on the quality of the interaction network, so missing or incorrect data can reduce their accuracy. Recent advancements in deep learning and multi-omics data integration help to overcome the computational challenges posed by high-throughput molecular data, enabling the identification of known and novel driver genes.

**FI-Net** [120] was introduced by Gu et al. to identify cancer driver genes by estimating the functional impact of somatic mutations. Functional impact refers to the degree to which a mutation alters the biological function of a gene, particularly its effects on protein structure and activity. The mutation impact it is typically measured based on FIS score [121]. FIS is based on evolutionary conservation patterns and calculated from multiple sequence alignment. FI-Net uses a feed-forward neural network to model functional impact scores from multi-omics features. The model was trained and validated on 31 cancer types from The Cancer Genome Atlas (TCGA), utilizing genetic, epigenetic, and transcriptomic data. FI-Net groups genes based on multi-omics similarity using hierarchical clustering and fits gamma distributions within each cluster to estimate background functional impact distributions. Genes are then statistically assessed for significant deviation from this background to identify drivers. FI-net demonstrated strong performance in identifying cancer driver genes across TCGA cancer types. Its effectiveness was evaluated based on overlap with known driver gene databases (CGC and NCG) and mutation impact metrics. It achieved high average precision, with 53.0% of predicted genes found in CGC and 88.2% in NCG. The identified genes also exhibited a high average deleterious mutation ratio (0.84) and broad mutation coverage (0.84). Notably, FI-net also revealed potential novel driver genes. Among them, GLI2 has been shown to be upregulated in benign tumors and ovarian cancer tissues [122], and it regulates surviving isoform expression in ovarian cancer [123]. The other six genes—GPR98, ZFYVE26, AHNAK2, APOB, ZNF236, and ODZ1—have also been associated with cancer in previous studies [124,125,126]

Schulte-Sasse et al. developed **EMOGI** [127], an explainable deep learning method that utilizes Graph Convolutional Networks (GCNs) to predict cancer genes by integrating multi-omics data (mutations, DNA methylation, gene expression) and Protein–Protein Interaction (PPI) networks. EMOGI expands the definition of cancer genes, acknowledging that they can be altered through mutations, copy number changes, or epigenetic mechanisms like promoter DNA methylation. The method was trained on pan-cancer data using the TCGA dataset. To enhance interpretability, EMOGI incorporates Layer-wise Relevance Propagation (LRP) [128] to explain individual predictions by identifying key features, such as mutations or interactions within PPI networks. LRP is an explainability technique used for deep neural networks that works by propagating prediction scores backward through the network layers to determine how much each input feature contributes to the final prediction. EMOGI successfully identified diverse genetic alterations in well-established cancer genes, including APC high mutation frequency in colon cancer, TWIST1 promoter hypermethylation, and MYC copy number amplifications across multiple cancer types. Across different PPI networks, on a test set of known cancer and non-cancer genes that were held out during model training, EMOGI demonstrated an average AUPRC of 71%, resulting in the best performing methods compared to popular methods in the literature [116,129,130]. The model was also tested on a never seen set of genes from OncoKB and ONGene. In these datasets, EMOGI had the best performance, but it showed, like all the other methods, a substantial drop in performance. This can be explained by the fact that these cancer gene sets from OncoKB and ONGene are compiled from either the scientific literature or clinical studies and therefore are not explicitly informed by any of the data types used to train EMOGI.

On the pan-cancer analysis, the authors identified 165 novel potential cancer genes (NPCGs) by aggregating the top-100 EMOGI predictions across six PPI networks and excluding previously known cancer genes. They showed that NPCGs are highly connected to known cancer genes in the protein interaction network, with classification driven mainly by network features. Furthermore, many NPCGs were found to be essential for tumor cell survival but are not simply housekeeping genes, indicating cancer-specific functional roles.

**MTGCN** [131] was implemented by Peng et al. to improve cancer driver genes identification. MTGCN’s key innovation lies in the Chebyshev graph convolution network [132] with the integration of biological features and structural information from Protein–Protein Interaction (PPI) networks to enhance gene representations like the EMOGI model. Furthermore, MTGCN adopts a multitask learning strategy that performs both node classification—identifying cancer driver genes—and link prediction—modeling protein–protein interactions—simultaneously. This approach enables the model to capture both the individual functional roles of genes and their interactions within biological networks. MTCGN was evaluated in a held-out test set of genes and compared with other state-of-the-art tools. It demonstrated impressive performance, ranking among the best methods alongside EMOGI, with a slight but consistent improvement over it. MTCGN was also evaluated on the independent gene sets from OncoKB and ONGene. Once again, MTCGN emerged as the top-performing tool, despite exhibiting the same drop in performance as EMOGI and other methods.

On the pan-cancer analysis, novel candidate genes were identified by MTGCN. The authors combined cancer pathway enrichment analysis and co-citation analysis with cancer-related terms (e.g., cancer, driver, tumor, biomarker, drug target) in the scientific literature. This approach helps prioritize genes that not only emerge computationally but also show supporting evidence in biological pathways and published research.

Using this framework, MTGCN was applied to unlabeled genes to explore its capacity for discovering new pan-cancer driver candidates. The resulting gene set shows strong enrichment in cancer-associated pathways, including small cell lung cancer, thyroid hormone signaling, and viral carcinogenesis. Several genes that are ranked low by other models—such as SYNE1, FLG, GRB2, MED13, and IRAK1—are highly prioritized by MTGCN and exhibit meaningful biological relevance. In particular, IRAK1 demonstrates strong co-citation with cancer-related literature and is involved in immune signaling pathways, suggesting its potential as a novel biomarker or therapeutic target.

**HGDC** [133] is a graph diffusion convolutional network that uses a graph diffusion technique to create a network specifically designed to capture similarities between nodes in a biomolecular network. This results in an auxiliary network that complements the original one. Both networks are then combined to learn a latent representation of the genes. Finally, a layer-wise attention classifier predicts the probability of each gene being a driver gene. What sets HGDC apart from models like MTCGC and EMOGI is that it does not rely on the assumption of network homophily. In simple terms, homophily means that connected nodes in a graph tend to share similar features and often belong to the same class. Traditional Graph Neural Networks (GNNs) generally perform well in homophilic settings but struggle when the network is heterophilic, where connected nodes may have different properties or labels. HGDC overcomes this limitation by using graph diffusion through a personalized PageRank algorithm, which is more effective in modeling relationships between driver and non-driver genes. In thorough comparisons with other approaches, the authors demonstrate that HGDC—thanks to graph diffusion and personalized PageRank—consistently outperforms both traditional methods and other GNN-based models, such as EMOGI and MTCGN. By integrating multi-omics data with the structural information of biomolecular networks, HGDC is not only able to discover new candidate cancer genes but also to identify driver genes at a patient-specific level.

A recent innovative approach, **DeepAlloDriver** [134], leverages deep learning techniques by adapting an equivariant Transformer [135] architecture combined with multi-head attention-weighted graph neural networks (EGNNs), specifically designed to handle graph-based data structures. DeepAlloDriver was developed to identify driver mutations at allosteric and potential allosteric sites. Previous studies, such as those by Shen et al. [136] and Tee et al. [137], have shown that deleterious mutations identified in cancer genomes are significantly enriched at protein allosteric sites. These studies propose that single-nucleotide polymorphisms (SNPs) may function allosterically, and that mutations at critical positions within the protein sequence could disrupt protein function through allosteric mechanisms. DeepAlloDriver analyzes protein sequences, structural information, and mutational data to predict whether a given mutation is likely to be a driver or passenger. The model was evaluated using 5-fold cross-validation. It achieved 94.1% accuracy, 93.8% precision, 94.3% recall, 93.9% specificity, and a 94.1% F1-score in detecting allosteric driver mutations, demonstrating the strong effectiveness of the method.

The authors also provided a valuable resource through a web server that predicts, based on gene symbols and amino acid substitutions, the probability of a mutation being a driver, aiding in the identification of potential therapeutic targets.

**GenomeBert** [138], a self-attention-based pretrained language model, was recently introduced for somatic driver mutation identification. The model was first pretrained on the human reference genome to obtain a robust understanding of genome sequences. Following this pretraining, GenomeBert was fine-tuned in a multitask learning framework, utilizing data from key cancer-related databases, including the Cancer Gene Census (CGC) [139], released by the Catalog of Somatic Mutations in Cancer (COSMIC), which provides a detailed list of oncogenes and tumor suppressor genes (TSGs). This multitask learning allows GenomeBert to simultaneously predict both oncogenes and TSGs, enhancing its ability to identify mutations in genes critical to cancer development.

For the somatic driver mutation identification task, the fine-tuned GenomeBert extracts hidden features from both the reference and altered sequences for each mutation. Then, the relative difference between these two hidden feature representations was extracted and used to train an XGBoost model to predict whether the mutation is a driver or a passenger. This step improves the classification of mutations by utilizing representations learned by GenomeBert during pretraining and fine-tuning.

Four key databases were employed to train and evaluate the model: Oncokb [140], FASMIC [141], and the xenograft experiment dataset [142] and PMID25348012 [143]. This combination of experimentally validated data ensures that the model is trained on highly accurate and reliable mutation information, enhancing the overall performance of GenomeBert in predicting somatic driver mutations. In a comparison with other tools, GenomeBert achieved state-of-the-art performance, showing slightly better results than DeepAlloDriver. However, a direct comparison with the other tools discussed in this section is not provided in the original paper. Overall, we emphasize that the aim of this review is not to benchmark the tools quantitatively but rather to highlight their innovations and qualitative performance. Therefore, although we report accuracy metrics and often refer to methods as state-of-the-art, these terms should be interpreted with caution, as the validation datasets and evaluation criteria often differ from one study to another.

A summary of the methods for cancer driver prediction can be found in Table 2.

### 4.3. Multi-Omics Survival Analysis

Survival analysis has become an essential tool for investigating time-to-event outcomes in biomedical research. In the context of multi-omic studies, it offers a powerful framework to explore associations between complex molecular profiles and patient survival. With the increasing availability of large-scale multi-omics datasets, it is now possible to model how different layers of biological information relate to clinical outcomes over time. Researchers can identify molecular signatures predictive of prognosis, stratify patients based on risk, and gain insights into the underlying biology driving disease progression. This analytical approach is particularly suited for integrative studies, where time-to-event data can be linked with heterogeneous omic features to improve understanding and support clinical decision-making.

**DeepOmix** [145] is a deep learning framework that integrates gene expression, DNA methylation, copy number variation (CNV), and gene mutation data to extract interpretable relationships across multi-omics layers. The authors designed a feed-forward neural network with a Cox proportional hazards layer to effectively model time-to-event outcomes while handling censored data. By leveraging the high-dimensional nature of multi-omics inputs, DeepOmix demonstrate better performance compared to traditional survival models in both risk prediction and patient stratification. DeepOmix was evaluated on eight different tumor types from TCGA using a repeated 10-fold cross-validation approach, where 90% of the data were used for model training and tuning, and the remaining 10% were used for testing. It outperformed five state-of-the-art methods including block forest [146], DeepHIT [147], and DeepSurv [148] in six out of eight tumor types, achieving an average concordance index (C-index) of 0.69, highlighting its predictive performance in survival analysis.

A key innovation of DeepOmix is the incorporation of prior biological knowledge through a functional module layer, which captures meaningful low-dimensional representations. Since genes operate together within regulatory networks, this layer reflects the biological reality that gene functions often performed. The functional module layer can include tissue-specific networks, gene co-expression networks, or established signaling pathways. This design not only enhances model interpretability but also facilitates the identification of significant modules associated with prognostic outcomes.

**MMOSurv** [149] is a meta-learning framework developed to tackle the challenge of survival prediction for rare cancers using only a few training samples. The model is built on a deep Cox proportional hazards framework and integrates gene expression and microRNA expression data using two parallel networks. These networks map different omics modalities into a shared dimensional latent space, aligned via a similarity loss. This allows MMOSurv to capture shared biological patterns and improve generalization in few-shot settings. It uses the Reptile [150] meta-learning algorithm to learn robust parameter initialization from multiple TCGA cancer types and quickly fine-tunes on the target cancer with only 10–20 samples.

MMOSurv was evaluated on nine cancer types in few-shot settings (10 and 20 samples) and consistently outperformed single-omics meta-learning in C-index and AUC metrics. It ranked highest across datasets and showed notable gains over pretraining and multitask learning, particularly for BRCA, COAD, and ESCA cancer types. By leveraging cross-omics similarities, MMOSurv learned patient-specific patterns from limited data, reaching an average C-index of 0.67. Remarkably, with just 10–20 samples, it matched or exceeded the performance of direct learning using up to 200 samples. Further comparisons with adapted few-shot versions of SurvCNN [151], DSM [152], and LAD-Network [153]—three state-of-the-art survival models—showed that MMOSurv consistently achieved higher C-index values. While these methods perform well on large datasets, their performance drops in low-sample settings. In contrast, MMOSurv leveraged cross-omics correlations via similarity constraints to maintain robust predictive accuracy, confirming its advantage in few-shot multi-omics survival analysis.

**MODeepHRD** [154] is a deep learning model for predicting homologous recombination deficiency (HRD) in gynecological cancers using multi-omics data. What distinguishes this approach is that it initially predicts HRD before conducting survival analysis. It integrates RNA-seq, miRNA-seq, DNA methylation, and somatic mutation data through a convolutional attention autoencoder, capturing both omics-specific and cross-omics representations. The model was trained on 351 ovarian cancer samples and validated across 2133 samples from 22 independent datasets, demonstrating robust generalization.

To address limited training data, MODeepHRD used a GAN-based data augmentation techniques to generate realistic synthetic samples and improve model robustness. MODeepHRD showed impressive performance on ovarian cancer, achieving an AUC of 0.88, F1-score of 0.89, and specificity of 0.90 using multi-omics data on HRD detection. In the external validation across 2070 ovarian cancer samples, MODeepHRD-predicted HRDpos tumor patients had significantly better survival and higher response to platinum therapy. The model also generalized well to BRCA and UCEC, identifying HRD positive tumors with improved survival and treatment benefit, independent of BRCA mutation status, thus confirming that MODeepHRD effectively leverages multi-omics data and data augmentation to accurately predict HRD status, enabling improved survival prediction and treatment response assessment across multiple gynecological cancers.

Chen et al. developed **PORPOISE** [155], a multimodal deep learning framework designed to integrate histopathological whole-slide images (WSIs) and genomic data for pan-cancer prognosis. Unlike models focused solely on molecular data, PORPOISE uses both tissue morphology and molecular profiles—mutations, RNA expression, and copy-number variation—to improve outcome prediction across 14 cancer types from TCGA, involving over 5700 patients and 6500 WSIs. PORPOISE is among the first models to perform survival analysis by extracting prognostic information directly from whole slide images (WSIs). It achieved an average concordance index (c-index) of 0.64 across 14 different cancer types. The model architecture comprises three key components: an attention-based multiple instance learning (MIL) network for processing WSIs, a self-normalizing neural network for omics data, and a fusion layer that integrates both modalities using a Kronecker product to capture cross-modal interactions. A major advantage of the attention-based MIL approach is its interpretability—it generates attention maps derived from attention scores, projected back onto the WSIs, along with attribution techniques to identify the most prognostically relevant image regions and molecular features. This interpretable design enables the discovery of meaningful histology-genomic links, like immune-rich regions correlating with better outcomes, making PORPOISE a valuable tool for both biomarker discovery and precision oncology.

Another important tool is **AUTOSurv** [156]. This is a deep learning framework developed for accurate and interpretable cancer survival prediction by integrating clinical data, gene expression, and miRNA expression data. AUTOSurv addresses the challenges of high-dimensional omics data and nonlinear predictor–outcome relationships through two modules: KL-PMVAE and LFSurv. KL-PMVAE is a pathway-informed variational autoencoder that reduces data dimensionality while capturing biologically relevant interactions. LFSurv is a multilayer perceptron predicting the Prognostic Index (PI) from both latent features and clinical variables. The “entangle” integration strategy in KL-PMVAE jointly learns gene and miRNA representations, enhancing performance over simple concatenation. KL-annealing is applied during training to stabilize latent feature learning. The model was trained and validated on TCGA breast (BRCA) and ovarian (OV) cancer datasets, using mRNA, miRNA, and clinical data. AUTOSurv outperformed traditional and deep learning baselines. AUTOSurv consistently demonstrated strong predictive performance in the multi-omics scenario (“mRNA + miRNA + clinical”) across several cancer types and datasets. In breast cancer (TCGA-BRCA), AUTOSurv achieved an internal median C-index of 0.75 and generalized well externally on the Caldas 2007 Breast Cancer dataset [157] with a C-index of 0.71, outperforming classical machine learning methods such as CoxPH-ENet, Random Survival Forest (RSF), extreme gradient boosting, and modified-SALMON [24]. For ovarian cancer (TCGA-OV), it reached an internal C-index of 0.63 and maintained the same on the ICGC-OVAU dataset with a C-index of 0.62. Moreover, AUTOSurv showed notable improvements when integrating multi-omics data over single-omics inputs, which was not consistently observed in modified-SALMON or other baseline methods. Similar trends were observed in other datasets, confirming AUTOSurv’s robustness and superior ability to fuse omics and clinical information for survival prediction across different cancers and cohorts.

**VAE-Surv** [158] is a multimodal deep learning framework designed for patient stratification and prognostic prediction in myelodysplastic syndromes (MDS). It integrates genetic, cytogenetic, and clinical data using a Variational Autoencoder (VAE) combined with a deep Cox proportional hazards model (DeepSurv). The VAE component reduces high-dimensional molecular data into a compact latent space, emphasizing local coherence and capturing nonlinear interactions among molecular markers. Clustering in this latent representation using K-means enables the identification of genetically distinct patient subgroups. Tested on the Genomed4all cohort of 2043 MDS patients and externally validated on a cohort of 2384 patients (IWG-PM cohort), VAE-Surv outperformed traditional CoxPH, and the identified clusters showed clear biological relevance, reflecting and refining the current World Health Organization (WHO) 2016 MDS classification. Although developed specifically for the MDS use case, the VAE-Surv framework is directly generalizable and can be applied to other biomedical contexts involving multimodal data. Within the MDS setting, VAE-Surv obtained a median C-Index of 0.78 in cross-validation and 0.74 on an external test cohort, outperforming both traditional CoxPH models and the survival cluster analysis approach [159]. Furthermore, the model revealed a more granular and biologically interpretable structure, delineating nine distinct patient clusters, compared to the three clusters obtained by other methods.

A recent study by Yang et al. [160] introduced a foundation model called **BEPH**, designed to extract rich representations from histopathological whole slide images (WSIs) using a Transformer-based architecture. Building upon this, the authors developed the **ClamSurvival** framework, a deep learning model tailored for cancer diagnosis and survival prediction. ClamSurvival leverages features extracted by BEPH and employs self-supervised learning to improve the accuracy and generalizability of prognostic predictions from WSIs. A distinguishing aspect of this work lies in the use of the BEiT-based Transformer backbone within BEPH, which enables the model to capture complex morphological patterns in pathology images. ClamSurvival is fine-tuned on labeled datasets spanning various cancer types and is capable of performing multiple tasks, including patch-level cancer diagnosis, WSI-level classification, and survival prediction across diverse cancer subtypes. Importantly, the authors benchmarked the features extracted using BEPH within ClamSurvival against those derived from several existing foundation models, demonstrating the superior performance of BEPH in downstream predictive tasks. The model achieved a concordance index (c-index) of 0.65 across six tumor types, highlighting its potential for integrating WSIs with clinical information to enhance cancer prognosis.

A summary of the methods for survival can be found in Table 3.

### 4.4. Drug Response Prediction

Recent advances in Drug Response Prediction (DRP) have been fueled by the availability of large-scale pharmacogenomic datasets. Among these, CCLE (24 drugs, 479 cell lines) [161], GDSC1 (320 drugs, 988 cell lines) [162], GDSC2 (175 drugs, 810 cell lines), and CTRP [163] (CTRPv1 with 354 drugs, 242 cell lines and CTRPv2 with 481 drugs, 860 cell lines) are the most widely used resources. Another notable resource is the PRISM dataset, which includes viability profiles for 4518 compounds across 578 cell lines obtained via pooled screening [164]. For CCLE and GDSC, extensive multi-omics data—including RNA-seq, methylation, proteomics, copy number variation, and comprehensive genomic variant call data from whole-exome, whole-genome, and targeted gene sequencing—enable robust characterization of cancer cell lines. As a result, many computational methods for drug response prediction try to integrate these diverse omic layers to generate rich representations of both cell lines and drugs, with expression data typically forming the primary basis and mutation data following closely [165].

To address the limitations of prior models based on handcrafted drug features or single-omics data, **DeepCDR** [166] proposes a hybrid framework that combines graph-based drug representations with multi-omics cell line profiles for drug response prediction. Drugs are represented as molecular graphs and processed by a graph convolutional network (GCN), which models atomic-level topological features such as atom types, valence, hybridization state, and local connectivity patterns to capture the structural context of each compound. On the cell line side, DeepCDR integrates genomic mutations, gene expression, and DNA methylation data through dedicated subnetworks, and it is evaluated on large-scale pharmacogenomic datasets including GDSC, CCLE, and TCGA.

Expanding on graph-based drug encoding, **GraphDRP** [167] presents a graph-based framework for drug response prediction that evaluates multiple graph neural network architectures—GCN, GAT, GIN, and a hybrid GCN-GAT—on molecular graphs of drugs. A key strength of the work lies in its comparative analysis of these models, each offering different strategies for capturing molecular topology. Cell lines are represented as binary vectors of genomic aberrations (mutations and copy number alterations), which are transformed using 1D convolutional layers. While the model focuses solely on genomic aberrations, excluding other omics such as gene expression or methylation, it incorporates saliency maps to enhance interpretability by highlighting the most influential genomic features in drug response prediction. GraphDRP was evaluated solely on the GDSC dataset.

Another approach based on graph modeling, **GraphCDR** [168], proposes a contrastive learning framework on a heterogeneous graph where nodes represent either drugs or cell lines and edges denote sensitive drug responses. A GNN encoder is applied to learn the latent embeddings of the drugs, then the drug response predictions are performed via an inner product operation. The contrastive task enhances generalization by comparing sensitivity–response graphs with corrupted ones based on resistant responses. It integrates mutations, gene expression, and methylation as cell-line features and molecular graphs for drugs. GraphCDR is evaluated on the GDSC and CCLE datasets, showing good performance and inductive capability.

A different strategy is adopted by **DeepTTA** [169], which replaces graph convolution with Transformer-based architectures to encode drug structures. It tokenizes SMILES strings into substructure sequences using the Explainable Substructure Partition Fingerprint (ESPF) algorithm, followed by a Transformer encoder that captures contextual and positional information. In parallel, gene expression profiles are processed through a FFNN. The key novelty lies in leveraging Transformer-derived drug embeddings in combination with transcriptomic data for IC50 prediction.

To overcome the over-smoothing effect and the limitations of modeling drugs and cell lines separately, **GADRP** [170] integrates multi-omics profiles (gene expression, methylation, CNV, and miRNA) and drug descriptors via an autoencoder-based dimensionality reduction, constructing a drug–cell line pair (DCP) network. The model introduces an Initial Residual and Layer Attention-based GCN (ILGCN), which preserves multi-scale neighborhood information through residual connections and adaptive layer-wise attention. Additionally, a K-nearest neighbors sparsification strategy is applied to reduce graph density and enhance the modeling of similarity-based interactions in high-dimensional heterogeneous data.

To improve generalization and robustness across both in vitro and in vivo drug response prediction settings, **MTIGCN** [171] proposes a unified multitask learning framework that simultaneously models drug sensitivity classification, IC50 regression, and similarity network reconstruction. It combines gene expression profiles of cell lines and molecular fingerprints of drugs within a graph-based architecture that enables the joint embedding of drug–cell line pairs. A notable feature of MTIGCN is its demonstrated transferability across domains: the model is trained on in vitro datasets (GDSC, CCLE) and successfully generalizes to in vivo settings, including PDX and TCGA.

The most common prediction target is the IC50—representing the concentration required to inhibit 50% of the cell population [165]—although the area under the dose–response curve (AUC) is also frequently employed and is considered preferable in several studies [172,173,174,175]. Model performance is typically evaluated using metrics such as Pearson correlation and RMSE, computed on K-fold cross-validation or over repeated train–test splits to obtain a robust distribution of results. In line with standard practices in drug response prediction (DRP), model evaluation can adopt increasingly stringent data splitting strategies: random (mixed-set) splits, where test samples are randomly selected drug–cell line pairs; unseen cell lines, where models are tested on entirely new cell lines; unseen drugs, where the test set includes drugs not seen during training; and unseen cell line–drug pairs, the most challenging setting, where both drugs and cell lines are novel to the model. These scenarios test different aspects of generalization, from interpolation within known entities to extrapolation toward unseen biological and chemical contexts [176]. Recent studies have highlighted that predictive performances can be inflated—particularly due to variance being driven by the drugs—emphasizing the need for tailored evaluation procedures to ensure a more reliable and realistic assessment of model generalizability [175,177,178].

**Figure 7:**
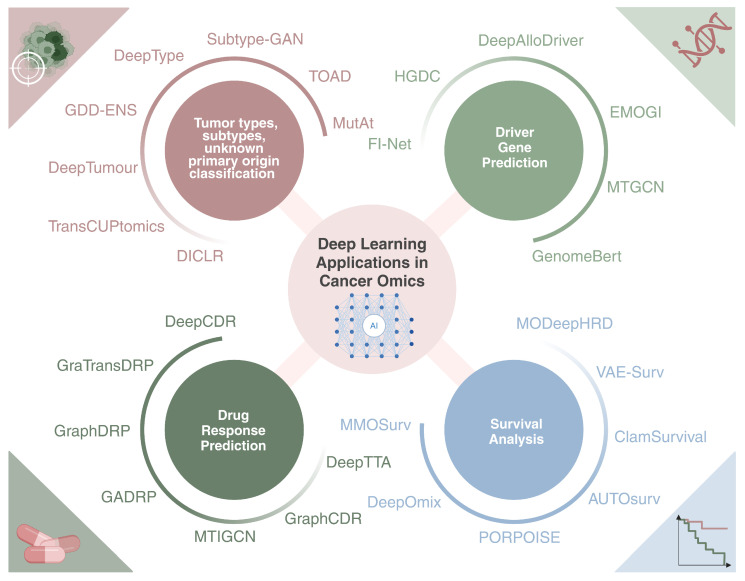
**Deep learning applications across key cancer omics tasks**. Summary of major deep learning tasks in cancer omics—including tumor classification, driver gene prediction, drug response prediction, and survival analysis—alongside representative models for each.

A summary of the methods for drug response prediction can be found in Table 4. Finally a visual summary of deep learning applications in cancer omics are depicted in Figure 7.

## 5. Conclusions

In this paper, we provided a comprehensive overview of recent advances in deep learning applied to cancer research using multi-omics data. While machine learning has become increasingly popular and accessible—thanks to a growing ecosystem of tools and libraries—it remains crucial to follow fundamental principles and best practices to avoid bias and ensure reproducibility. For this reason, we proposed a basic roadmap of machine learning aimed at a broad and interdisciplinary audience.

We also emphasize that, as multi-omics and deep learning continue to gain attention and popularity, the establishment of standardized practices and benchmarking criteria is essential and in some cases still lacking. This would empower researchers and clinicians to select appropriate tools based on their specific needs. Another critical consideration is explainability. It is good practice—particularly in the development of future methods—to always keep in mind that the ultimate goal of these tools should be to advance their applicability in clinical settings. Until we achieve a deeper understanding of how these models operate and can ensure that they are reliable, robust, and trustworthy, their integration into clinical workflows will remain cautious and gradual.

In summary, this review highlights key developments in the application of deep learning to cancer genomics, with specific focus on cancer type classification, driver gene identification, drug response prediction, and survival analysis—offering a comprehensive snapshot of recent trends and techniques in the cancer genomics field.

## Figures and Tables

**Table 1:** **Summary of deep learning methods for classification of tumor types, subtypes, and unknown primary origin**. GEx = gene expression, DNAm = DNA methylation, SNVs = single-nucleotide variants, CNV = copy number variations, SV = structural variants, FFNN = feed-forward neural network, GAN = generative adversarial network, TRNN = Transformer neural network, VAE = variational autoencoder, RF = random forest, CUP= cancer of unknown primary origin, HMF = Hartwig Medical Foundation [106], HPA = Human Protein Atlas [108], BWH = Brigham and Women’s Hospital [32].

Model	Architecture	Task	Data Type	Strength	Possible Limitations	Datasets
DeepType (https://github.com/runpuchen/DeepType, accessed on 23 May 2025) [87]	FFNN	Tumor subtyping	GEx	Supervised classification with K-means clustering	Single omics data. No. of cancer types	METABRIC (BRCA), TCGA (BLCA)
Subtype-GAN (https://github.com/haiyang1986/Subtype-GAN, accessed on 23 May 2025) [93]	GAN	Tumor subtyping	CNVs, DNAm, miRNA, mRNA	Multiomics data. Adversarial learning with GMM clustering	High-dimensional data. No of cancer types	TCGA (10 cancer types)
DICLR (https://github.com/ykxhs/DILCR, accessed on 23 May 2025) [95]	Custom VAE	Tumor subtyping	DNAm, miRNA, mRNA	Multiomics data. Noise disentanglement. Self-supervised clustering	High-dimensional data. No. of cancer types	TCGA (10 cancer types)
MuAt (https://github.com/primasanjaya/mutation-attention, accessed on 23 May 2025) [99]	TRNN	Tumor typing, subtyping, and CUP	SNVs/MNVs, indels, SVs	Many mutational events. Attention mechanism. No. of cancer types	High-dimensional data. Single omics	PCAWG, TCGA (24 cancer types)
GDD-ENS (https://github.com/mmdarmofal/GDD_ENS, accessed on 23 May 2025) [102]	FFNN	Tumor typing and CUP	Targeted sequencing	Ensemble neural network. Gene panel	Standard neural network architecture	MSK-IMPACT (38 cancer types)
TransCUP-tomics [104]	VAE + RF	CUP	GEx	No. of cancer types	Single omics. No code available.	TCGA (39 cancer types), GTEx, HPA
DeepTumour (https://github.com/ICGC-TCGA-PanCancer/TumorType-WGS, accessed on 23 May 2025) [105]	FFNN	CUP	SNVs, Indels, CNVs, SVs	Many mutational events. Passenger mutations. No. of cancer types	Single omics	PCAWG (24 cancer types), HMF
TOAD (https://github.com/mahmoodlab/TOAD, accessed on 23 May 2025) [32]	CNN encoder + attention modules	CUP and tumor status	H&E images	Weakly supervised instance learning. Attention mechanism. Multitask learning	Single omics	BWH (18 cancer types), TCGA

**Table 2:** **Summary of deep learning methods for cancer driver prediction**. GEx = gene expression, DNAm = DNA methylation, SNVs = single-nucleotide variants, CNV = copy number variations, PPI = protein–protein interaction, GGI = gene–gene interaction, GCN = graph convolutional neural network, EGNN = equivariant graph neural network, LLM = large language model, XGB = XGBoost, LRP = layerwise relevance propagation, NCG = network of cancer genes [144], CGC = Cancer gene Census.

Model	Architecture	Task	Data Type	Strength	Possible Limitations	Datasets
FI-Net [120]	FFNN	Cancer drivers	Mutations, DNAm, GEx, HiC	FIS-score. No. of cancer types. Multiomics data.	High-dimensional data, standard neural network architecture. Not evaluated on an independent test set of genes	TCGA (31 cancer types), NCG, CGC
EMOGI (https://github.com/schulter/EMOGI, accessed on 23 May 2025) [127]	GCNs	Cancer drivers	Mutations, DNAm, GEx, PPI network	LRP for explainability. Multi-omics data	High-dimensional data	TCGA (16 cancer types), NCG
MTGCN (https://github.com/haiyang1986/Subtype-GAN, accessed on 23 May 2025) [131]	GCN	Cancer drivers and link prediction in PPI network	Mutations, DNAm, GEx, PPI network	Chebyshev GCN. Multi-omics data. Multitask learning	High-dimensional data	TCGA, NCG, KEGG, OncoKB
HGDC (https://github.com/NWPU-903PR/HGDC, accessed on 23 May 2025) [133]	Diffusion GCN with PPR	Cancer drivers	Mutations, DNAm, GEx, PPI, and GGI networks	Diffusion process. Multi-omics data. Can deal with heterophilic networks	High-dimensional data	TCGA, KEGG, Reactom, GGNet, PPNet
DeepAlloDriver (https://mdl.shsmu.edu.cn/DeepAlloDriver, accessed on 23 May 2025) [134]	EGNN	Cancer drivers on allosteric sites	Gene symbol and amino acid substitution	Equivariant architecture. Attention mechanism. Prediction on allosteric sites. Web Server	-	RCSB Protein Data Bank, Allosteric Database
GenomeBert (https://github.com/GaryinDeep/GenomeBert, accessed on 23 May 2025) [138]	LLM + XGB	Oncogene prediction, tumor suppressor genes and cancer drivers	DNA sequences	LLM for feature extraction	Interpretability. Computationally expensive.	GRC, CGC, OncoKB, FASMIC

**Table 3:** **Summary of deep learning models for survival analysis.** GEx = gene expression, DNAm = DNA methylation, CNV = copy number variation, miRNA = microRNA, CL = clinical, WSIs = whole-slide images, TCGA = The Cancer Genome Atlas, GEO = gene expression omnibus, ICGC = International Cancer Genome Consortium, SCAN-B = Sweden Cancerome Analysis Network-Breast.

Model	Architecture	Task	Data Type	Strength	Possible Limitations	Datasets
DeepOmix [145]	FFNN	Risk prediction and patient stratification	CNV, DNAm, GEx, Mutations	Functional module layer and cox proportional hazard layer	No code available	TCGA
MMOSurv (https://github.com/LiminLi-xjtu/MMOSurv, accessed on 23 May 2025) [149]	FFNN	Risk prediction	GEx, miRNA	Meta-learning for few-shot learning	No external validation dataset	TCGA
MODeepHRD (https://github.com/ZhouSunLab-Workshops/MODeepHRD, accessed on 23 May 2025) [154]	Convolutional Attention Mechanism	HRD classification and prognosis prediction	miRNA, DNAm, RNA-seq, mutations	GAN-based augmentation. Survival based on the HRD status	Synthetic data can lead to bias	ArrayExpress, ICGC, TCGA, GEO, SCAN-B
PORPOISE (https://github.com/mahmoodlab/PORPOISE, accessed on 23 May 2025) [155]	MIL + FFNN	Time bin classification	WSIs, mutations, CNV, RNA-seq	Multi-omics data. Explainability. WSI images.	Prediction of time bins. Foundation model based on ResNet-50 (obsolete)	TCGA
AUTOSurv (https://github.com/jianglindong93/AUTOSurv, accessed on 23 May 2025) [156]	VAE + FFNN	Prognostic index	CL, miRNA, GEx	VAE and CL	High-dimensional data	Caldas-BC, ICGC, TCGA
VAE-Surv (https://github.com/compbiomed-unito/VAE-Surv, accessed on 23 May 2025) [158]	VAE + DeepSurv	Risk prediction and patient stratification	CL, Mutations, cytogenetic alterations	Unified, genetic patient clustering and survival prediction	Tested only on a specific use case	GenoMed4All, IWG-PM MDS cohorts
ClamSurvival (https://github.com/Zhcyoung/BEPH, accessed on 23 May 2025) [160]	Attention + FFNN	Risk prediction	WSIs, CL	Multitask learning. WSI images.	-	TCGA, BreakHis, LC25000, NCT-CRC-HE-100K, CAMELYON16, BACH

**Table 4:** **Summary of deep learning models for drug response prediction.** GEx = gene expression, DNAm = DNA methylation, SNPs = single-nucleotide polymorphisms, CNV = copy number variation, DMG = drug molecular graphs, ESPF = explainable substructure partition fingerprint, DFPs = drug fingerprints, ILGCN = initial residual and layer-attention based graph convolutional network, MTL = multi-task learning, DS = drug sensitivity, PDX = patient-derived xenografts, TCGA = The Cancer Genome Atlas.

Model	Architecture	Task	Data Type	Strength	Possible Limitations	Datasets
DeepCDR (https://github.com/kimmo1019/DeepCDR, accessed on 23 May 2025) [166]	CNN + GCN	IC50 regression and DS classification	SNPs, GEx, DNAm, DMG	Multi-omics integration; validation on TCGA	-	GDSC, CCLE, TCGA
GraphDRP (https://github.com/hauldhut/GraphDRP, accessed on 23 May 2025) [167]	CNN + GNN	IC50 regression	SNPs, CNV, DMG	Comparative GNN evaluation; interpretable via saliency maps	Limited to binary genomic features; single dataset	GDSC
GraphCDR (https://github.com/hauldhut/GraphDRP, accessed on 23 May 2025) [168]	GNN + contrastive learning	DS classification	GEx, SNPs, DNAm, DMG	Heterogeneous graph; contrastive learning; multi-omics integration	-	GDSC, CCLE
DeepTTA (https://github.com/CZenkert/DeepTTA-implementation, accessed on 23 May 2025) [169]	Transformer + FFNN	IC50 regression and DS classification	GEx, Drug SMILES substructures (ESPF)	Transformer-based drug encoding; interpretable substructures	Only GEx omics data; single dataset	GDSC
GADRP (https://github.com/flora619/GADRP, accessed on 23 May 2025) [170]	Autoencoder + ILGCN	IC50 regression	GEx, DNAm, CNV, miRNA, DFPs	Residual GCN; multi-omics; PRISM data	-	PRISM, CCLE
MTIGCN (https://github.com/weiba/MTIGCN, accessed on 23 May 2025) [171]	GNN + MTL	IC50 regression and DS classification	GEx, DFPs	Multi-task learning; in vivo evaluation	No drug structure modeling	GDSC, CCLE, PDX, TCGA

## Data Availability

No new data were created or analyzed in this study. Data sharing is not applicable to this article.
